# Stress as Offense to Self: a Promising Approach Comes of Age

**DOI:** 10.1007/s41542-019-00041-5

**Published:** 2019-07-01

**Authors:** Norbert K. Semmer, Franziska Tschan, Nicola Jacobshagen, Terry A. Beehr, Achim Elfering, Wolfgang Kälin, Laurenz L. Meier

**Affiliations:** 1 Department of Psychology, University of Bern, Fabrikstr. 8, 3012 Bern, Switzerland; 2 Institut de Psychologie du Travail et des Organisations, University of Neuchâtel, Rue Emile-Argand 11, 2000 Neuchâtel, Switzerland; 3 Central Michigan University, Sloan Hall 233, Mount Pleasant, MI 48859 USA

**Keywords:** Self-esteem, Offense to self, Illegitimate tasks, illegitimate stressors, appreciation, subtle offense, Boosts to the self

## Abstract

Stress is related to goals being thwarted. Arguably, protecting one’s self, both in terms of personal self-esteem and in terms of social self-esteem, is among the most prominent goals people pursue. Although this line of thought is hardly disputed, it does not play the prominent role in occupational health psychology that we think it deserves. Stress-as-Offense-to-Self theory focuses on threats and boosts to the self as important aspects of stressful, and resourceful, experiences at work. Within this framework we have developed the new concepts of illegitimate tasks and illegitimate stressors; we have investigated appreciation as a construct in its own right, rather than as part of larger constructs such as social support; and we propose that the threshold for noticing implications for the self in one’s surroundings typically is low, implying that even subtle negative cues are likely to be appraised as offending, as exemplified by the concept of subtly offending feedback. Updating the first publication of the SOS concept, the current paper presents its theoretical rationale as well as research conducted so far. Research has covered a variety of phenomena, but the emphasis has been (a) on illegitimate tasks, which now can be considered as an established stressor, and (b) on appreciation, showing its importance in general and as a core element of social support. Furthermore, we discuss implications for further research as well as practical implications of an approach that is organized around threats and boosts to the self, thus complementing approaches that are organized around specific conditions or behaviors.

“Issues pertaining to the self are ubiquitous at work.” (Ferris et al. 2018, p. 1)

In this paper, we present and update the theory of Stress as Offense to Self (SOS; Semmer et al. 2007, Semmer et al. 2016, and we summarize more than ten years of research conducted in the context of this concept. In a nutshell, SOS is based on the assumption that achieving and maintaining a positive self-view is a basic need and that threats to self-esteem generate strain (i.e., negative psychological, physical, and behavioral reactions; Beehr and Glazer 2005), whereas boosts to self-esteem foster well-being. While the basic assumption is not new, the importance of a positive self-view and its relationship to health-related outcomes should receive more attention in occupational health psychology to a greater degree than is currently the case. SOS theory was developed in the context of research on occupational stress; this tradition is reflected in the preponderance of negative aspects (threats) as opposed to positive aspects (boosts). However, although often “bad is stronger than good” (Baumeister et al. 2001; Sinclair et al. 2015), stress cannot be understood without considering resources (Bakker and Demerouti 2017; Hobfoll 2001), and the theory also includes a focus on resources that are relevant to the self, notably appreciation.

This paper has three parts. We first discuss concepts in psychology (not only occupational health psychology) that constitute important foundations of SOS theory and show the basic importance of threats and boosts to the self. We also discuss to what extent theories in occupational health psychology refer to aspects related to the self, concluding that these aspects have largely been treated in a peripheral way. In the second part, we present SOS theory in more detail and discuss empirical research conducted in this context. In the final part, we present an outlook in terms of open questions, research necessities, and practical implications.

## Basic Concepts

### Stress as Thwarting Important Goals

Probably the most common denominator of stress definitions is that stress thwarts, or threatens to thwart, important needs and goals. Thus, Lazarus (1999, p. 70) stated that “without a goal at stake, there is no potential for stress or emotion”. Similarly, Carver and Connor-Smith (2010) state: “It may be, however, that stress is nothing more (and nothing less) than the experience of encountering or anticipating adversity in one’s goal-related efforts” (pp. 683–684).

The extent to which events and circumstances are conducive to people’s goals and the needs behind these goals is a central component in appraisal theories (Ellsworth and Scherer 2003), which are an important element of most occupational stress models (Spector and Goh 2001). Being inherent in many work situations, goals should play an especially prominent role in occupational stress. Empirically, the positive affective consequences of attaining goals, or moving towards them, are well established, as are the negative affective consequences of not attaining, or moving away from, goals (Plemmons and Weiss 2013). Given the importance of goals for the stress process, it is somewhat surprising that theories in the field of occupational stress, while acknowledging the role of goals in an abstract way, typically do not specifically focus on goals and do not specify *which* goals are especially important. Rather, as Elliot et al. (2011) note, “goals represent the ground rather than the figure in the stress and coping literature” (p. 644).

One approach that does focus on goal content, albeit in a very general way, distinguishes between approach and avoidance goals and examines different characteristics of stress experiences, emotions, and coping associated with these two basic goal characteristics (e.g., Carver and Connor-Smith 2010; Carver et al. 2008). Furthermore, self-determination theory (SDT) focuses on specific content, postulating that autonomy, competence and relatedness constitute basic psychological needs (Deci et al. 2017; Van den Broeck et al. 2008). Basic Psychological Needs Satisfaction (BPNS) fosters autonomous motivation, which, in turn, results in a number of positive consequences, including well-being (Reis et al. 2000). Weinstein and Ryan (2011) conclude that “consistent deprivation of needs is considered a cumulative risk factor for stress incursion and poor stress response” (p. 12), and this conclusion is affirmed meta-analytically by Van den Broeck et al. (2016).

Some other theories in occupational health psychology contain more or less explicit references to goals or needs. Many authors emphasize the role of control as a resource (Karasek 1979; Karasek and Theorell 1990; Spector 1998), and some argue that control not only has instrumental value (e.g., facilitating coping) but also satisfies a *need for control* (Frese 1989; Semmer and Beehr 2014), an interpretation that is in line with self-determination theory. However, this aspect has not played a prominent role in occupational health psychology.

Thus, although the role of goals and needs for stress and well-being often is acknowledged, these aspects are not the focus of occupational health psychology. In contrast, the current article focuses on a need that we believe is very important and has a pervasive influence on the experience of stress and its implications for health and well-being: the need to preserve and enhance a positive evaluation of oneself, or the need for self-esteem.

### Maintaining Self-Esteem as an Important Goal

There is great consensus in the literature that people are motivated to feel good about themselves (Alicke and Sedikides 2009). Not all approaches see self-esteem itself as an inherent need, but rather as a warning system regarding a thwarted need to belong (sociometer theory; Leary and Baumeister 2000) or as a shield against the fear of death (Greenberg and Arndt 2012); for an overview see Brown and Zeigler-Hill (2018). However, virtually all agree that people are pervasively concerned about self-esteem and that threats to self-esteem are stressful and provoke attempts to enhance and protect it (Alicke and Sedikides 2009; Steele 1988). This basic concern for self-esteem is a central aspect of SOS theory, which argues that boosts and threats to self-esteem are topics that deserve more attention in occupational health psychology. Two topics regarding self-esteem are especially relevant in our context: a global sense of self-esteem versus self-esteem related to professional identity, and personal versus social self-esteem.

#### Global Self-Esteem Versus Self-Esteem Related to Professional Identities

People occupy social roles, and they often identify with them to a considerable degree (Katz and Kahn 1978; Ryan and Deci 2012). Of specific importance for our context is that professional roles tend to become part of people’s identity (Ashforth and Schinoff 2016; Haslam and Ellemers 2005) and, thus, part of the self (Oyserman et al. 2012). People often have high esteem for their professional roles; they defend them against threats (Gollwitzer et al. 2013), and they regard them favorably when comparing them to other professions (Meyer et al. 2006). As a result, people react in a positive way to experiences that affirm their professional role (e.g., with pride and enhanced self-esteem); conversely, threats to their professional roles tend to induce stress (Cast and Burke 2002; Stets 2005; Warr 2007). As the SOS concept focuses on the occupational realm, this implies that aspects of the professional role should be considered with regard to the need for self-esteem. An analogous reasoning applies to self-esteem tied to being a respected member of an organization rather than a specific profession (organization-based self esteem or OBSE; Pierce and Gardner 2004; Pierce et al. 1989).

#### Personal Versus Social Self-Esteem

In line with Lazarus (1999) or Rubin and Hewstone (1998), we distinguish between personal self-esteem and social self-esteem. *Personal* self-esteem refers to one’s self-evaluation in terms of aspired qualities (e.g., competence, attractiveness, honesty), but also intrinsic qualities such as the dignity one possesses as a human being. Note that personal does not mean “a-social”. Internal, personal standards that are experienced as the individual’s private standards are likely to be internalized social standards (Kaplan 2006), which may become somewhat independent of social judgements of others; Leary et al. (2009) speak of private self-esteem. In contrast, *social* self-esteem refers to the degree to which one feels esteemed, acknowledged, and appreciated by significant others; thus, it depends strongly on one’s social surrounding. Obviously, the two facets are not independent of one another. The distinction between personal and social self-esteem is reflected in the two parts of SOS theory: stress based on self-evaluation and stress based on evaluation by others.

### Self-Esteem in Ocupational Health Psychology

The SOS concept emphasizes that threats to personal as well as social self-esteem at work are important triggers of stress. In the following, we take a closer look at the role established theories of occupational health psychology attribute to these aspects. We first discuss “classic” models, which do not specifically focus on the self: the job demand-control model and its extension, the job demands-resources model, the conservation of resources model, and role stress theory. We then discuss models that attribute a more important role to the self: justice/fairness, effort-reward imbalance, and challenge-hindrance stressors. We also discuss interpersonal stressors; for them, no established theory exists, but they represent a classic threat to the self and therefore deserve special attention. We focus on the extent to which threats and boosts to the self are treated prominently in these models.

As the models deal with stressors and resources, we should mention that we define “stressors” in a probabilistic sense, that is, as events or conditions that increase the risk of a reaction in terms of strain. The emphasis on risk is important, as not everyone reacts to the same event or condition in the same way (Lazarus 1999). However, in a given population, one can identify events and conditions that are associated with an increased risk, analogous to risk factors in epidemiology (Semmer et al. 2005; Spector and Goh 2001). Regarding resources, we follow Hobfoll (2001, p. 339), who defines resources as “those objects, personal characteristics, conditions, or energies that are valued in their own right, or that are valued because they act as conduits to the achievement or protection of valued resources”; as for stressors, we emphasize the probabilistic nature of this definition.

#### Job Demand-Control, Job Demands-Resources, Conservation of Resources, and Role Stress Models

Four models have been especially influential in organizational health psychology: The Job Demand-Control model and its extension, the Job Demands-Resources model, the Conservation of Resources model, and the Role Stress model. We will briefly review these models in terms of the way they deal with threats to the self.

*The Job Demand-Control model* (JDC; Karasek 1979) has largely focused on demands in terms of workload/time pressure. Aspects potentially related to self-threat, for example conflicts, were explicitly mentioned as demands in Karasek’s original paper (Karasek 1979, p. 287), and social support, another variable strongly linked to self-esteem, was added later (see Karasek and Theorell 1990). Nevertheless, there is no strong focus on issues related to self-esteem in this model or in the research inspired by it.

The extension of the JDC model, the *Job Demands-Resources (JDR) model* (Bakker and Demerouti 2017) includes a large number of demands and resources; variables relating to self-esteem, such as appreciation, have been investigated in the context of this model as important resources (Bakker et al. 2007). Nevertheless, issues of self-esteem are not in the focus of the JDR model; the same is true for the *Conservation of Resources (COR) model* (Hobfoll 2001), although self-esteem is classified as one of several key resources (Halbesleben et al. 2014).

*Role Stress Theory* is a very early model (Kahn et al. 1964) that has generated a great amount of research (Beehr and Glazer 2005; Kahn and Byosiere 1992). Focusing on expectations from various sources, it refers to clarity of expectations (role ambiguity), to compatibility between different expectations (role conflict) and to discrepancies between expectations and resources (role overload). Already in 1964, Kahn et al. mention a loss of self-confidence as a frequent consequence of stress; this aspect is, however, not in the focus of the model.

#### Models of Organizational Justice, Effort-Reward Imbalance, and Challenge-Hindrance Stressors

Several theories focus more strongly on aspects related to self-esteem: justice/fairness (we use these terms interchangeably), the Effort-Reward Imbalance model (ERI), and the theory of challenge-hindrance stressors.

Research and theory on *organizational justice* have emphasized that being treated fairly signals acceptance and respect (De Cremer and Tyler 2005), an argument that has been made especially forcefully by Bies (2015), who regards human dignity as a core element of interactional justice. It therefore is not surprising that justice research has been connected to issues of stress and health (De Cremer and Tyler 2005; Greenberg 2010). Injustice has been shown to induce stress-related emotions (Mikula et al. 1998; Weiss et al. 1999) and to predict health outcomes, such as cardiovascular disease (De Vogli et al. 2007; Elovainio et al. 2006). The implications of justice for how accepted people feel make the field of justice/fairness an important area when studying stress as offense to the self.

*The Effort-Reward Imbalance concept* (ERI; Siegrist and Wahrendorf 2016) has many parallels with justice/fairness, as it emphasizes a fair social exchange. ERI treats self-esteem not only as an outcome variable, but also as an important element of the processes it postulates. Briefly, ERI postulates that health and well-being depend on the degree to which effort and rewards are balanced. Efforts refer to classic demands, much in the tradition of the JDC model (Karasek 1979), with workload and time pressure being prominent; rewards refer to financial rewards, status rewards (career possibilities; job security) and socio-emotional rewards in terms of esteem and recognition. Research on ERI typically does not determine the specific impact of esteem reward, which is most important in the context of the current paper; results by Van Vegchel et al. (2002) indicate, however, that esteem reward may exert a particularly strong effect.

Due to its connection to goals, the concept of *challenge and hindrance stressors* also is related to self-esteem. Hindrance stressors, such as performance constraints, present obstacles to goal attainment and are postulated to have only negative consequences. By contrast, challenge stressors, such as time pressure, although associated with negative outcomes, may simultaneously have positive consequences; these are typically described in terms of gains, chances for learning and development, or goal advancement (Crawford et al. 2010). Such consequences can be regarded as affirming the self (Rodell and Judge 2009; Widmer et al. 2012), although self-esteem is not typically a central part of the concept and the pertinent research. Arguing for a central role of self-esteem with regard to challenge stressors, Widmer et al. (2012) showed that time pressure (a typical challenge stressor) was related to organization-based self-esteem, which mediated the the association between time pressure and a positive attitude towards life.

#### Interpersonal Stressors and Leadership

According to Sonnentag and Frese (2013, p. 562), *interpersonal, or social, stressors* refer to “poor social interactions with direct supervisors, coworkers, and others”… but also include “interpersonal conflicts at work, (sexual) harassment, mobbing or bullying, and other kinds of workplace aggression” (see also Hershcovis 2011; Nielsen et al. 2016). The core of such stressors is “relational devaluation” (Leary et al. 2006), as illustrated by such behaviors as insults, condescending behavior, and ridicule (Hershcovis 2011).

Although not necessarily described specifically in terms of threats to the self, the importance of the behaviors discussed for the self runs through these publications – reflected, for instance, in the use of terms such as insulting, demeaning, or being put down (e.g., Cortina et al. 2001; Hershcovis 2011). Andersson and Pearson (1999) regard a perceived “identity threat” as the “tipping point” that induces incivility spirals (p. 462).

Furthermore, threats to self-esteem are a recurrent topic in the literature on *social exclusion* at work (ostracism; Williams and Nida 2011). Involving withholding positive attention, ostracism is distinct from incivility and similar behaviors that involve allocating negative attention; yet the two are related in that they “breach expectations of mutual respect” (Ferris et al. 2017, p. 328) and thus constitute an offense to one’s social esteem (Pereira et al. 2013). In general, however, although threats to the self are present at least implicitly, the literature on aggression / rejection / exclusion is not specifically organized around threats to the self but rather around specific behaviors (e.g., incivility, aggression, rejection).

On the positive side, *social support* – a core resource in occupational health psychology – includes a component of being valued and esteemed (Beehr 2014; Semmer et al. 2008), and Dormann and Zapf (2004) discuss customer gratefulness as a source of self-esteem. We will come back to the issue of social support later.

Research on *leadership* has emphasized qualities that imply acknowledgment and appreciation (e.g., consideration, support), and thus foster a positive self-concept (Montano et al. 2017; Zwingmann et al. 2014); on the negative side, destructive leadership “is perceived as hostile and/or obstructive” (Schyns and Schilling 2013, p. 141), thus presenting a threat to self-esteem. More recent concepts emphasize the expression of appreciation and respect more explicitly (Van Quaquebeke and Eckloff 2010). Although threats to self-esteem (or related concepts) are mentioned, or implied, they are not a central part of these approaches.

#### Threats to the Self in Occupational Health Psychology: Summary

In sum, although the role of self-esteem is recognized in justice theories (Bies 2015; Greenberg 2010) and in ERI (Siegrist and Wahrendorf 2016), threats (and boosts) to self-esteem do not constitute a central focus of most prominent approaches in occupational health psychology. Self-esteem is mostly confined to being investigated as a predictor variable, an outcome variable, or a moderator variable (Ganster and Schaubroeck 1991; Keller et al. 2015; Kuster et al. 2013; Mäkikangas and Kinnunen 2003); sometimes as part of the broader core self-evaluation construct (Judge 2009); sometimes as a more focused, work-related construct (organization-based self-esteem; Pierce and Gardner 2004). Largely missing, however, is self-esteem as an exploratory construct that helps understand many stressful experiences and their impact. Given the importance of the self for employee well-being, threats (and boosts) to the self deserve a more systematic treatment. SOS theory can contribute to filling this gap. It will be presented next.

## The “Stress-as-Offense-to-Self” Approach

We contend that implications for the self of conditions at work deserve to occupy a more prominent role in occupational health pychology. Given the importance of self-esteem in people’s lives, we assume that threats and boosts to self-esteem are involved in many stressful as well as resourceful episodes and conditions; and we have started to delinate possible implications of this assumption theoretically, and to test them empirically. More specifically, we assume that aspects relevant for the self are not confined to obvious acts of disrespect, as in aggression (Neuman and Baron 2005) or in relationship-focused conflict (Weingart et al. 2015); rather, they are ubiquitous in everyday (working) life. Thus, going beyond obvious social messages, we look at implications for self-esteem of phenomena such as job design and common stressors. On the positive side, we assume that boosts to the self, such as appreciation, are important resources. We further assume that, given the importance of maintaining a favorable self-view, people are vigilant to aspects related to the self, reacting not only to conspicious aspects but also to rather subtle cues (Bargh 1982; Gray et al. 2004; Humphreys and Sui 2015). As we distinguish between personal self-esteem and social self-esteem, we organize our theory around these two constructs and focus on threats, and boosts, to the personal as well as to the social self. The two aspects are strongly related; characteristics that are personally important often are characteristics that are important for social acceptance as well (Leary and Allen 2011).

Stress for the *personal self* relates to the degree to which one meets one’s personal standards for one’s behavior and performance, implying that one can think of oneself as a capable, dependable, highly performing, and honorable individual. Obviously, such criteria are social in nature, but personal self-esteem is affected only to the degree that individuals have internalized such standards and accept them for themselves. There are two topics in occupational health psychology that are especially relevant for personal self-esteem: performance in terms of success and failure, and moral aspects related to one’s own work such as being honest, not exploiting others, being dependable. If the criteria for good performance or adequate behavior are not met, one feels insufficient; which is why we refer to this aspect as *Stress through Insufficiency* (SIN). Note that (in)adequate performance by itself is not enough to make these effects occur; the person in question must also attribute this to internal causes (Weiner 2014), which results in self-relevant emotions such as shame and guilt in the negative, and pride in the positive case (Pekrun and Perry 2014; Tangney and Tracy 2012).

With regard to the *social self*, we speak of *Stress as Disrespect* (SAD). SAD refers to the extent that one feels appreciated, accepted, and valued versus derogated, treated unfairly, ignored, attacked, or excluded. Such experiences likely result in a number of negative emotions such as hurt, sadness, and anger in the negative case (Smart Richman and Leary 2009), whereas feeling accepted and valued is associated with positive feelings and emotions, such as contentment and pride. As mentioned above, and exemplified below, (not) being appreciated can originate in interpersonal behavior, but also in conditions at work, such as job design, and stressors and resources. Table 1 summarizes the model.

**Table 1:** Aspects of work likely to affect self-relevant emotions and self-esteem

Stress as Offense to Self (SOS)	
Self-Evaluation: **Stress through Insufficiency (SIN)**	Evaluation by Others: **Stress as Disrespect (SAD)**
(In) adequate performance + internal attribution	Messages of (Dis-)Respect contained in
(In) adequate moral behavior	• Interpersonal behavior
	• Work design
	• Task assignments (e.g., illegitimate tasks)
	• Stressful conditions and events (e.g., illegitimate stressors)

SOS theory has a number of implications. Some of these implications relate to the importance and ubiquity of phenomena that are well-known but not taken seriously enough in much research and theorizing, such as appreciation; some relate to thresholds, such as subtly offending feedback; some relate to new phenomena, such as illegitimate tasks, or illegitimate stressors. In the following, we will present the concepts and phenomena we have investigated in this context. Rather than simply following the logic of the model, as presented in Table 1, however, we will start with the two areas in which most of the research has been conducted: The concept of illegitimate tasks, and the role of appreciation at work. This part will be followed by further research along the logic of the model, that is, SIN and SAD.

### Research Focus # 1: Illegitimate Tasks

Implications for self-esteem often seem obvious in the case of interpersonal behavior, such as bullying, or incivility; this is less the case for conditions at work, notably for job design, although these can also be assumed to affect self-esteem. The way tasks and jobs are designed can convey messages that threaten or boost the self. Regarding job design, the focus has traditionally been on performance, motivation, and satisfaction (Hackman and Oldham 1980; Humphrey et al. 2007), more recently also on basic psychological needs (Deci et al. 2017), but not primarily on self-esteem.^1^ It was Pierce and Gardner (2004; Pierce et al. 1989) who put self-esteem at the center of their approach, fousing on organization-based self-esteem (OBSE). They describe that many features of job design, such as complexity and autonomy, are associated with OBSE, and these associations have been confirmed meta-analytically (Bowling et al. 2010). In addition, many organizational variables (e.g., mechanistically versus organically designed social systems), organizational messages of being valued such as trust or fairness, and an environment that facilitates task performance, have been shown to be associated with OBSE (Pierce and Gardner 2004); Bowling et al. (2010) report associations between a variety of stressors and resources and OBSE. The sociologists Kohn and Schooler (1973) have demonstrated that “occupational self-direction”, characterized by “freedom from close supervision, substantively complex work, and a non-routinized flow of work” (p. 97) are associated with self-esteem (see also Mortimer and Finch 1986). Similarly, in a recent multi-wave study, Keller et al. (2015) showed that high quality jobs, characterized by high autonomy and skill variety, predicted self-esteem, albeit only for men; for women, self-esteem predicted high quality jobs, indicating that women need self-esteem to overcome the barriers for attaining high quality jobs.

Altogether, there is quite some evidence that conditions at work are associated with self-esteem, although research on this issue is still sparse. The aspects investigated in these studies are mostly those that are established in the job-design literature, such as autonomy and complexity (Bowling et al. 2010); these are important, but there are reasons to go beyond these variables. Pierce and Gardner (2004, p. 593) note that self-esteem may be influenced not only by “messages sent from significant others in one’s social environment” but also from “the implicit signals sent by the environmental structures to which one is exposed.” From an SOS perspective, it is thus necessary to look for such implications beyond well established job design variables. More specifically, we have developed a new stressor concept, called *illegitimate tasks*, which can be considered a stressor that predicts well-being over and above more traditional variables.

#### The Concept of Illegitimate Tasks and its Measurement

As noted above, people tend to value their professional identity (Ashforth and Schinoff 2016; Haslam and Ellemers 2005; Meyer et al. 2006), which implies that conditions and events that affirm one’s professional identity represent a boost to self-esteem, whereas threats to this identity are perceived as stressful (Cast and Burke 2002; Gollwitzer et al. 2013; Stets 2005; Warr 2007).

Asking what constitutes this professional identity leads to the issue of *core* tasks: Professions are defined by characteristic activities. Teachers teach, doctors heal, and welders weld. Sometimes the core tasks refer not to specific activities but rather to responsibilities, as when police officers define their profession as enforcing the law, or managers see themselves as ensuring that their team does good work; such responsibilities may entail a great variety of different activities, as long as they relate to the goals inherent in their responsibilities. Thus, people holding a given job can be *expected* to do certain things or to deal with certain problems; this is the core of the role concept (Katz and Kahn 1978). To the extent that people identify with their professional role, carrying out activities that correspond to their core role can be assumed to affirm their identity (Ashforth and Schinoff 2016; Katz and Kahn 1978). Thus, Gabriel et al. (2011) showed that the effect of nurses’ satisfaction with successful task completion on affect after work was significantly stronger for “direct care” tasks (which correspond to core tasks) as compared to indirect tasks.

We have carried this line of thinking a step further by looking at the reverse of role expectations: If employees in a given position can legitimately be expected to carry out specific activities, it follows that there must be activities that they *cannot* legitimately be expected to carry out. Normally, a cook is not expected to serve, and a waiter is not expected to cook. Note that the same activity may be part of the core role under certain circumstances but not others. For instance, when we asked nurses if it was stressful to have a patient who kept calling the nurses for all kinds of things (e.g., getting a cup of tea) on a day that was already stressful to begin with, many said something like “that’s not really stressful; after all, it’s part of the job!”. However, with the patient having regained strength and being able to carry out these activities him- or herself, the same scenario yielded completely different reactions, such as an indignant “we are not a hotel here!” (Semmer 2000). In the first case, the activity was considered supporting the healing process, which is at the core of the nursing role. In the second case, it was perceived as being treated like a maid rather than a nurse; this is a different role, not the role that the nurse takes pride in, and therefore the activity was resented.

In another interview study (Semmer et al. 2006) we asked participants to describe their daily tasks and to indicate which of these tasks they believe they should not have to do. The 159 participants reported 3548 tasks and sub-tasks; of these, they considered about one third as not legitimate. In line with the assumed importance of core tasks, tasks described as subsidiary tasks had a much higher chance to be seen as illegitimate (64% of 1559), as compared to core tasks (10% of 1989). Two types of tasks were appraised as illegitimate, which we termed unnecessary tasks and unreasonable tasks.

*Unnecessary tasks* are tasks that simply should not exist. They may exist because other people did not work correctly (e.g., having to manually transfer data from one computer system to another one because the two systems are incompatible) or because they are perceived as useless (e.g., having to write reports that people believe no one ever reads). Note that the term unnecessary may apply to problems that led to the task but occurred earlier; thus, the data transfer could have been avoided if management had assured compatibility when the systems were established; once two systems are set up, it may well be necessary to do the manual transfer. “Unnecessary” therefore refers to lack of justification for the task to have come into existence; it does not necessarily imply that carrying out the task *at a given moment* is not necessary. It is, however, considered a waste of time.

Whereas unnecessary tasks are illegitimate for anyone, *unreasonable tasks* are tasks that are not part of the role of specific employees; they should be done by someone else. Note that unreasonable tasks need not be beneath someone’s dignity, as in so-called “dirty work” (Kreiner et al. 2006). Waiters may consider it illegitimate to be asked to cook, even if they regard cooking as a perfectly honorable activity and also have the competence required; they just do not regard it as their job. Also, assigning a higher-status task may be illegitimate, as when a new resident is left alone with the responsibility for a whole ward at night. Thus, it is not the task *as such* that is illegitimate; it is its social meaning in relation to one’s work role. The example of the nurses given above illustrates this nicely: The very same activity may be reasonable when it is regarded as supporting the healing process, but as demeaning when it does not. As a result, the legitimacy of a task cannot be determined from the task alone; the specific circumstances that determine its meaning have to be taken into account. These circumstances are to a large degree defined by one’s role in the organization, which is why illegitimate tasks can be considered a special type of role conflict (Beehr and Glazer 2005), specifically as a type of person-role conflict (see Semmer et al. 2015). Furthermore, if employees choose such a task themselves (e.g., offering help), there is no illegitimacy. The task has to be assigned, and attributions are important in that the person assigning the tasks could, and should, have refrained from doing so (Cropanzano et al. 2001; Semmer et al. 2015).

To assess illegitimate tasks, the *Bern Illegitimate Tasks Scale* was developed (BITS; Jacobshagen 2006; Semmer et al. 2015). The *BITS* contains four items on unnecessary tasks; an example is “Do you have work tasks to take care of, which keep you wondering if they would not exist (or could be done with less effort), if things were organized differently?”. Four items refer to unreasonable tasks, for example, “Do you have work tasks to take care of, which you believe should be done by someone else?” Responses range from *never* (1) to *frequently* (5)*.* Internal consistency has repeatedly been found to be adequate, both for the two subscales and for the scale as a whole. Although the two sub-constructs are expected to correlate substantially, the construct of illegitimate tasks is not unidimensional. In such a situation, it is appropriate to focus either on the superordinate construct or on the sub-constructs (Bagozzi and Edwards 1998). Some authors have used the two subscales separately; in our studies, we have been focusing on the overall construct. In structural equation models, the construct is best represented by modeling illegitimate tasks as a latent construct with the two subscales as parcels (Semmer et al. 2015). Having only two parcels is possible if the measurement model includes other constructs as well (Kline 2005), and the two-indicator model has the advantage of explicitly modeling the dimensionality of the construct (Hall et al. 1999; Little et al. 2013).

#### Research on Illegitimate Tasks

The first paper on illegitimate tasks appeared in 2010 (Semmer et al. 2010). A number of studies have appeared since, and they confirm that illegitimate tasks are related to a variety of strains. These strains include *self-esteem* (Eatough et al. 2016; Schulte-Braucks et al. 2019; Semmer et al. 2015; Sonnentag and Lischetzke 2018) and job *identity* (Ma and Peng 2019); *behaviors* such as counterproductive work behavior (Semmer et al. 2010; Zhou et al. 2018) and sickness presenteeism (Thun et al. 2018); performance ratings by supervisors (Ma and Peng 2019; Meier and Semmer 2018); or slips, trips, and falls (Elfering et al. 2018). Strain associated with illegitimate tasks further includes behavioral *intentions* (intention to quit; Apostel et al. 2018); *affective strain* such as burnout, irritation, and feelings of resentment towards one’s organization (Semmer et al. 2015); *physiological* measures such as cortisol (Kottwitz et al. 2013); and *recovery*-related variables such as sleep quality (assessed by actigraphy; Pereira et al. 2014) and detachment (Sonnentag and Lischetzke 2018).

Research on illegitimate tasks has employed a variety of methodological aproaches. Some studies are cross-sectional, some are longitudinal. Most studies analyze between-person effects, but an increasing number also investigated within-person effects (Eatough et al. 2016; Kottwitz et al. 2013; Madsen et al. 2014; Pereira et al. 2014; Sonnentag and Lischetzke 2018; Zhou et al. 2018).

Studies analyzing unnecessary and unreasonable tasks separately tend to find stronger effects for unreasonable tasks (e.g., Pindek et al. 2018; Schmitt et al. 2015; Semmer et al. 2015), although not always (Meier and Semmer 2018). Theoretically, stronger associations for unreasonable tasks are to be expected, as they relate specifically to the focal person’s professional role, whereas unnecessary tasks are unnecessary for everyone. The different studies controlled for various variables that may potentially be confounding, most notably variables that are conceptually close, such as organizational justice or social stressors.

Overall, therefore, these studies confirm that illegitimate tasks can be considered a stressor that is uniquely associated with a variety of different stress-symptoms (strain). It is noteworthy that these symptoms include self-esteem, both on an inter-individual (Schulte-Braucks et al. 2019; Semmer et al. 2015; Sonnentag and Lischetzke 2018), and on an intra-individual level (Eatough et al. 2016; Schulte-Braucks et al. 2019; Sonnentag and Lischetzke 2018); this is in line with a core assumption of SOS theory, as is the finding by Ma and Peng (2019) that illegitimate tasks affected job identity. Furthermore, Mühlethaler et al. (2011) found that illegitimate tasks predicted lower levels of experienced success six and twelve months later, in line with the assumption that illegitimate tasks are unlikely to confirm one’s professional identity and thus make experienced success less likely.

In some cases, effects of illegitimate tasks were confined to boundary conditions, such as relatively poor values of perceived health (Kottwitz et al. 2013), appreciative leadership (Apostel et al. 2018), flexible role orientation (Ma and Peng 2019), or high values of time pressure (Zhou et al. 2018). Furthermore, in some cases illegitimate tasks moderated the effect of other stressors. Thus, Schmitt et al. (2015) found a curvilinear effect of time pressure on work engagement, indicating that time pressure acted as a challenge stressor (Crawford et al. 2010) up to a point; however, this positive effect of moderate time pressure on work engagement was found only when unreasonable tasks were low, whereas the association was linear when uneasonable tasks were high.

#### Subjectivity in Assessing Illegitimate Tasks and the Issue of Culture

Mostly, illegitimate tasks are assessed in terms of individual ratings, implying that differences between individuals are to be expected, both in terms of individual appraisals and in terms of differences in reactivity. Thus, Meier and Semmer (2018) found that supervisors reported a lower frequency for illegitimate tasks of their employees than the employees themselves did. Several studies reported effects of illegitimate tasks being moderated by individual differences, such as organizational ownership (Van Schie et al. 2014), justice sensitivity (Schulte-Braucks et al. 2019), or health status (Kottwitz et al. 2013; Madsen et al. 2014).

However, individual ratings are not simply idiosyncratic; they also reflect cultural communalities (see Rosso et al. 2010). Culture, defined as a “shared meaning system” (Erez and Gati 2004, p. 587) exists at many levels – “at regional and national levels, at the industry or institutional level, and at the organizational level. There are also occupational cultures and subcultures within organizations based on functions and tasks.” (Schein 2000, p. xxix). In line with this reasoning, Semmer et al. (1996) demonstrated communalities in appraisals for workers holding the same job, both for stressors and for well-being (their “shared job strain” variable is conceptually similar to the “shared burnout” variable in Moliner et al. 2005), which argues against attributing all differences between people to individual differences. Länsisalmi et al. (2000) report shared perceptions of stressors, coping strategies, and well-being in different organizational units; employing multilevel analyses. A number of studies have shown that aggregated perceptions of conditions at work can contribute to the prediction of outcomes such as well-being (Tucker et al. 2005), job satisfaction (Cambré et al. 2012), or burnout (Moliner et al. 2005); see Bliese and Jex (2002).

Regarding the dependence of illegitimate tasks on culture, it is important to note that the definition of illegitimate tasks is based on social norms (“I should not have to do this”). Differences are therefore to be expected not only at the level of individual differences but also on various cultural levels. In terms of occupations, the fact that some professions have a term for what we call illegitimate tasks (e.g., “non-nursing activities”; Sabo 1990) suggests that professional groups may have a shared idea of illegitimate tasks. In a similar vein, medical doctors frequently refer to an exceedingly high level of administrative tasks (Thun et al. 2018). Shared appraisals may well be different for specific subcultures; for instance, in hospitals that define themselves as mostly related to well-being (e.g., cosmetic surgery), nurses may perceive service as part of their proper job, whereas in more traditional hospitals they should more likely perceive such tasks as “non-nursing activities” (Semmer 2000). The occupation of software engineers seems to be developing from a “nerdy ‘lone wolf’ job” to a job requiring frequent communication and regulation of emotional display (Serebrenik 2017), so that the range of tasks considered legitimate for this profession may expand.

On a national level, Ahmed et al. (2018) demonstrate cultural differences regarding the effects of illegitimate tasks; they could confirm their model only in their US sample but not in an Indian sample (although there also were some differences in occupations). However, Munir et al. (2017) found relationships of illegitimate tasks with burnout and resentment in a sample of Pakistani teachers. The extent and type of such cultural differences remains to be examined.

The study by Meier and Semmer (2018), mentioned above, illustrates that the differences found between job incumbents and their supervisors are due not only to individual differences but are also influenced by the requirements of their specific occupational roles. Meier and Semmer (2018) based their hypotheses on their “roles-as-perspective” (RaP) theory. In brief, they postulate that roles influence the perception of stressful conditions, including illegitimate tasks, because the roles imply different perspectives: Supervisors have a broader *scope*; they are responsible for a unit and therefore likely to give greater weight to considerations concerning the unit as a whole, whereas incumbents are more likely to focus on their individual concerns. Furthermore, *attribution* processes are important. Supervisors are responsible for task assignments and therefore may have a tendency to downplay the amount of illegitimate tasks. Finally, the role of the supervisor implies a certain *distance*, which increases with the number of employees the supervisor is responsible for. Thus, Meier and Semmer (2018) propose that the leadership role implies certain (sub-)cultural communalities. In line with the theory, the authors found that supervisors reported fewer illegitimate tasks than incumbents. Furthermore, the correlation of *r* = .18 (*p* < .05) confirmed the expectation that convergence between supervisors and incumbents should be low to moderate. In line with the presumed effect of role distance, the number of subordinates moderated the extent of convergence, which was higher for supervisors with few, as compared to many, subordinates. Both incumbent and supervisor ratings of illegitimate tasks predicted three outcome variables: psychological strain (incumbent self-report of exhaustion), behavioral strain (supervisor report of incumbent incivility), and family strain (incumbent’s partner report of work-family conflict). These associations remained significant when the two subscales of illegitimate tasks were controlled for each other. These results suggest that supervisors and incumbents both have a concept of illegitimate tasks, and that their concepts are overlapping but not identical. The impact of individual differences and cultural differences at various levels certainly should be a focus of future research.

In sum, research related to illegitimate tasks is promising, although many questions remain, which necessitate further research. However, the basic quality of illegitimate tasks as a stressor can be considered well established by now.

### Research Focus # 2: Appreciation at Work

The most direct way to boost people’s self-esteem is to show that one appreciates them. Because appreciation conveys a clear and direct message about the self, it is allocated a central role as a resource in SOS theory. Although often described in different terms, appreciation is a central construct in pertinent social psychological theories, for instance in terms of messages that signal “relational value” (Leary and Allen 2011); it also is an important construct in theories of justice, notably interactional justice (Bies 2015). In the area of work and organizations, it is frequently mentioned but often investigated as an aspect of larger constructs rather than as a construct in its own right. For instance, in the leadership area, appreciation is seen as an aspect of consideration (Judge et al. 2004) or of “good leadership” (Kuoppala et al. 2008). As a notable exception, Van Quaquebeke and Eckloff (2010) put appreciation (which they refer to as respect), in the center of their respectful leadership approach. A recent study by Wang et al. (2018) specifically focused on “recognition events” and demonstrated the effects of appreciation at work in the morning on afternoon work engagement, mediated by positive affect but also by relatedness-need satisfaction and competence-need satisfaction as specified by SDT (Deci et al. 2017).

There is considerable overlap between appreciation and social support, including perceived organizational support (Kurtessis et al. 2017). Social support has been an important concept in research on (occupational) stress and well-being for a long time, and it has been linked to self-esteem, notably through its emotional component, which includes communicating esteem (Beehr 2014; Sarason et al. 1996; Thoits 2011). The question therefore arises if appreciation is worth studying as a construct in its own right that is important beyond social support. We will argue that appreciation is not just one of many elements of social support but rather a core element that is essential for the positive effects of support to occur. It therefore deserves to be studied as a separate construct because it conveys a boost to the self in a very direct way.

#### Appreciation as a Construct in its Own Right

In a first, cross-sectional study (Stocker et al. 2010), we demonstrated that appreciation was associated with higher job satisfaction and lower feelings of resentment. Especially important for our argument that appreciation is not simply part of other constructs (which are considered to be the really important ones) but a construct in its own right, these effects held over and above not only the “classic” resource of job control but also over and above two conceptually related variables, social support and interactional justice.

In a diary study (Stocker et al. 2014), multilevel analysis showed that events reported to involve appreciation from several sources (supervisors, colleagues, and customers/clients) predicted serenity in the evening, thus indicating a positive effect on recovery from stressful demands at work, which is of central importance for preventing long-term effects (Geurts and Sonnentag 2006).

A content-analysis of the situations described showed that expressing praise and gratitude was by far the most frequent way of showing appreciation (52%); other categories involved expressing trust (e.g., by asking employees about their opinion) and granting responsibility (e.g., by assigning a challenging task), showing support and respect, declaring that one enjoyed working with the person, and tangible rewards and promotion.

In a two-wave study, Stocker et al. (2018) showed that appreciation by supervisors buffered the effects of interruptions at work on job satisfaction, self-efficacy, job-related depressive mood, and sleep problems. These results held when controlling for time pressure and job control, but also when controlling for social support.

#### Appreciation and Social Support

Reviewers of manuscripts on appreciation often ask if we are not actually measuring social support. It therefore is noteworthy that two of the three studies mentioned above controlled for social support, and the effects of appreciation held. From an SOS perspective, one may even go a step further and argue that appreciation is a core element of social support. This idea is not new; pioneers of research on social support argued long ago that “the essence of a supportive relationship is the communication of acceptance and love” (Sarason et al. 1996, p. 21). However, for quite some time the discussion has focused on different types of support, especially instrumental and emotional support (Beehr 2014), asking which type of support is most useful under what circumstances (Cutrona and Russell 1990). Semmer et al. (2008) argued that appreciation, which implies communicating esteem, is not only a central component of emotional support but actually a core element of social support in general, *including instrumental* support. In an interview study, we asked participants to describe a situation in which they received support, and coded the situations as reflecting instrumental or emotional support. We also asked why this support was helpful, again coding the responses as instrumental or emotional. Around 60% of the situations described were instrumental; however, for only about 28% of these the support was perceived as helpful due to its instrumental value. By contrast, for 71% of instrumental support situations the helpful elements were described in terms of emotional support alone (49%) or in combination with instrumental reasons (22%). As an example, a person described that someone went grocery shopping for her because she was unable to move due to back pain. When asked why this was helpful, the answer was not having food in the house again (instrumental), but rather “You know, he invested two hours of his time to do this for me” (Semmer et al. 2008, p. 242), referring to the esteem aspect of social support.

These results raise the question of what happens if social support (of any type) lacks the esteem element. It is well established that social support can be threatening to self-esteem if it signals a lack of competence or resilience of the recipient (Beehr et al. 2010; Fisher et al. 1982). Semmer et al. (2018b) developed a questionnaire measuring *dysfunctional social support* in the sense of receiving instrumental support that is not accompanied by communicating esteem. Items refer to perceiving the support as being delivered reluctantly, not taking the problem seriously, or making premature suggestions. The analyses showed that social support proper was related to lower strain, but dysfunctional social support was related to higher strain.

#### Summary

There are good reasons to regard appreciation as a construct that deserves to be taken seriously in its own right. Rather than “just” being an element of other constructs, such as social support or consideration in leadership, it may well be the core element of these constructs in terms of health effects. Thus, supportive acts without esteem do not simply lack positive effects – they actually may be conceived as stressors. Such a perspective is practically important because many people may not be fully aware of the signals they send, or fail to send, while providing support. Thus, many people may be convinced to be supportive when offering quick suggestions as to how to solve a problem (“You only have to…”). Despite being well-intended, such suggestions may communicate that the problem the focal person is facing is easy to solve, thus making it difficult to understand why the focal person would struggle with that problem and implying that the focal person is overly sensitive, lacks resilience, or is unable to cope in an effective way. Again, many of these behaviors involve small-scale, often subtle signals rather than “big events”.

The fact that rather subtle and small events are involved, and that dysfunctional support may not be recognized as such by those who provide it, may be one reason why appreciation is not abundant in organizations. Thus, the frequency of 0.9 events involving appreciation per day reported by Stocker et al. (2014) certainly does not indicate a well developed culture of appreciation. Indeed, not recognizing employee achievements ranks top among complaints of employees about their leaders (Solomon 2015). Even the very basic issue of listening attentively often poses problems (Van Quaquebeke and Felps 2018), and leaders sometimes are reluctant to praise employees for work well done (Elfering 2016). Thus, it is important to raise awareness of the importance of appreciation, of the many – and often subtle – ways to show it, but also of the many – and, again, often subtle – ways of neglecting the appreciative component when delivering social support (see also the concept of sensitive support; Feeney and Collins 2015). These considerations apply to interpersonal exchanges (e.g., involving supervisors or colleagues) as well as to organizational efforts to support employees in an appreciative way (perceived organizational support; Kurtessis et al. 2017).

### Further Concepts and Related Research: Stress through Insufficiency (SIN)

Blaming oneself and being proud of oneself are prototypical experiences with regard to self-esteem; they can be regarded as self-conscious emotions in that they “directly involve self-reflection and self-evaluation” (Tangney and Tracy 2012, p. 446). These emotions are strongly tied to achievement, and are therefore called achievement emotions by Pekrun and Perry (2014). Success and failure are central categories within the SIN-part of SOS theory. However, achievement is not the only aspect that is relevant; another one is moral adequacy (Meier et al. 2013; Tangney and Tracy 2012).

#### Success and Failure

Achievement is a core characteristic of work environments (Latham and Locke 2007; Zacher et al. 2016). It is almost axiomatic that achieving goals, or making progress towards goals, is a pleasurable experience (Carver and Connor-Smith 2010; Plemmons and Weiss 2013). Given that work is intrinsically tied to achieving task-related goals (Latham and Locke 2007; Zacher et al. 2016), one would expect a great amount of field research on this issue (Mühlethaler and Semmer 2017), but the number of pertinent studies is surprisingly low. Furthermore, studies in this area tend to focus on rather long-term goals that cover months or even years (Klug and Maier 2015). In contrast, there is rather little research on daily goal attainment (Gabriel et al. 2011; Harris et al. 2003; Holman et al. 2005; König et al. 2010). Furthermore, research tends to focus on goals set explicitly in advance. Workdays, however, are full of unpredicted events implying a need to revise goals or to set new goals, but also events implying opportunities to achieve goals that are not consciously salient all the time but are activated when opportunities arise (Kruglanski et al. 2002; Shah 2005). The study by Wang et al. (2018), already mentioned above, included such achievement events and showed that, like appreciation, they were associated with engagement, mediated by positive affect, competence-need satisfaction, and relatedness-need satisfaction. Within the SOS research program, we have begun to study goal attainment and failure, expecting the first to constitute a boost, the latter a threat to the self.

Studies on daily *goal attainment* showed that reports obtained after work on goal achievement during the work day, predicted positive affect at bedtime, which mediated an effect of goal achievement on job satisfaction next morning (Mühlethaler and Semmer 2017). Similarly, daily achievements predicted sleep quality reported next morning, mediated by serenity in the evening (Mühlethaler et al. 2018).

On the negative side, we investigated *daily failures*. We assumed that failures are not simply a stressor like any other (e.g., high workload); rather, because failures threaten one’s sense of being a competent individual, and thus one’s self, they should constitute a special stressor that has effects beyond that of other daily stressors. In a diary study, we asked participants to report daily negative events (stressors); for each of these events, we also asked if participants attributed them to their own performance. Over and above the basic stressfulness of the event, performance failure predicted self-relevant feelings of incompetence, guilt, and shame, and reduced the tendency to seek social support; these effects were mediated by blaming oneself (Semmer et al. 2018a). These studies support the notion that failure and success are special kinds of stressors and resources, because they are connected to the self.

#### Moral Behavior

Compared to performance, we have so far focused less on moral adequacy within SOS theory. However, Meier et al. (2013) reviewed literature on unethical behavior, and requests to perform such behavior, in relation to their negative health consequences for both targets and witnesses. Requests to perform unethical behaviors were mentioned in descriptions of illegitimate tasks provided by participants in a study by Pindek et al. (2018).

### Further Concepts and Related Research: Stress as Disrespect (SAD)

One of the tenets of SOS theory is that people’s thresholds for perceiving offending messages should be rather low, because the concern for maintaining self-esteem is so strong. One of the implications of this postulate is that phenomena that have been investigated in terms of rather strong offenses may also have effects at much lower intensity. In this context, we have investigated subtly offending feedback. A second concept we will present here takes up research on attribution and postulates that not only tasks can be illegitimate, but so can stressors.

#### Subtly Offending Feedback

Destructive feedback has long been recognized as offending people and inducing hurt feelings, and it is clearly related to the self. However, destructive feedback has mostly been operationalized in a rather extreme way (Raver et al. 2012), containing strongly offensive statements that were “general, inconsiderate in tone, attributed the poor performance to internal factors, and included threats” (Baron 1988, p. 200); similarly, descriptions in vignette-studies typically suggested an internal attribution unambiguously (Hareli and Hess 2008). However, there are many ways that feedback can direct attention to the self (Kluger and DeNisi 1996), and we contend that very subtle cues often will suffice, in accordance with the claim that people tend to be sensitive to messages concerning the self (Bargh 1982; Leary and Baumeister 2000). We therefore investigated “subtly offending feedback”, which is negative in content but delivered (through a video) in a friendly tone (as opposed to Baron’s more severe operationalization) and without explicitly suggesting internal attribution (as opposed to Hareli & Hess’s operationalization). Rather, the offending nature of the feedback was indirect; it was conveyed in three different ways: *Overkill* implied dwelling on mistakes, especially minor ones, at great length, continuously citing reasons why something was a mistake. The idea was that with every new “argument”, the mistake would appear more obvious and foreseeable, so that it becomes increasingly hard to understand why the actor could make such a mistake. *Exaggeration* referred to blowing up mistakes beyond measure, for instance by characterizing typos as something that made a paper worthless. *Banalizing* suggested that nothing was easier than avoiding a given mistake, thus making the actor appear either incompetent or completely unmotivated. The self-relevant conclusions were not stated explicitly, but insinuated by the three strategies. As expected, students observing a video with a professor giving such feedback to another student judged feedback fairness lower for subtly offending feedback than for constructive, but higher than for destructive feedback (Krings et al. 2015). An interview study (Krings et al. 2016) confirmed that employees do experience these kinds of feedback at work, and their answers could reliably be coded according to the three categories.

This research is important for two reasons. Theoretically, it supports the notion that only minimal cues are required to direct people’s attention to devaluative aspects of a communication (Leary and Baumeister 2000); this is in line with developments in the area of aggression / counterproductive behavior, which investigate incivility in terms of comparatively small, and often rather ambiguous, offenses (Cortina et al. 2017; Meier and Semmer 2013). In practical terms, subtle offenses may well happen without intention, and even without awareness. Supervisors may try to deliver feedback constructively but fail to notice the subtle offenses it contains, because they employ a friendly tone and do not suggest internal attributions explicitly. Notably the widespread recommendation that one should provide a rationale for negative feedback may induce people to provide too many reasons and thus induce overkill, rather than giving reasons in a sufficiently detailed way but not more, as proposed by the principle of “minimally invasive negative feedback” (Semmer and Jacobshagen 2010).

#### Illegitimate Stressors

Not only tasks can be considered as more or less legitimate, but so can stressors. In contrast to illegitimate tasks, which are not necessarily stressful by themselves, however, illegitimate stressors are stressors to begin with. Thus, illegitimate stressors should evoke stress-reactions even if they were not illegitimate; however, a high degree of illegitimacy should aggravate their impact.

What makes a stressor illegitimate? Again, the concept of core elements of a given role is important. Some stressors are inevitably tied to specific professions. For example, in a medical environment, professionals are expected to deal with emergencies even if they imply overtime; social workers must deal with people in difficult circumstances; prison guards must deal with potentially violent prisoners; teachers must deal with students who have a hard time understanding. Such experiences often are stressful, but at the same time they are a normal part of the job, implying that they are, at least to some degree, unavoidable. Job-specific stressors are tied to one’s professional identity, and thus should be experienced as less stressful than other stressors; that was found by Peeters et al. (1995) and by Haslam et al. (2005). Dealing with such stressors may even become a source of pride, signaling the endurance, skill, and accomplishment associated with one’s profession (Meara 1974). By contrast, illegitimate stressors are perceived as potentially avoidable. For example, a machine breakdown is likely to be stressful, but more so if employees see it as caused by the previous shift not working as diligently as they should have. As with illegitimate tasks, the appraisal implies an attribution suggesting that others could, and should, have acted differently (Cropanzano et al. 2001).

We are aware of only one study on illegitimate stressors so far. In an event-sampling study by Semmer et al. (2019), participants described stressful situations. For each situation, additional questions referred to the stressfulness of the situation but also to its perceived illegitimacy (e.g., unnecessary, inadmissible, avoidable). Beyond the initial stressfulness of the situation, illegitimacy predicted lower well-being, higher feelings of resentments towards one’s organization, threatened social esteem (e.g., feeling treated disrespectfully), and more desire for revenge. Research on illegitimate stressors has just started; the first evidence available suggests that this concept deserves more attention.

## Conclusions and Outlook

### Broader Implications of SOS Theory

#### Relevant Events and Circumstances

SOS theory focuses on the human concern for a positive self-image in term of personal and social self-esteem (Alicke and Sedikides 2009). We advocate a stronger focus on the many triggers for self-esteem to be threatened or boosted at work. Some of these triggers, such as social support or incivility, have received quite some attention in occupational health psychology but the connection to the self has not been a focus. Some triggers, such as appreciation, have mostly been investigated as parts of other constructs rather than as constructs of their own; some, such as success at work, have received attention in a limited way. Still others, such as illegitimate tasks and illegitimate stressors, are constructs newly developed within the SOS approach. In other words, SOS has suggested

- a fresh look on well-known phenomena that deserve more attention in occupational health psychology (e.g., success and failure; appreciation);
- a reconceptualization of well-established phenomena in term of threats or boosts to the self as a core element (e.g., social support);
- new constructs, such as illegitimate tasks and illegitimate stressors.

#### Relevant Outcomes

As the SOS approach is concerned with implications for the self, self-conscious emotions are important candidates for potential outcomes; they include emotions such as shame, guilt, and pride (Dickerson et al. 2009; Pekrun and Perry 2014; Tangney and Tracy 2012). Furthermore, effects on state self-esteem may occur (and have been found; Eatough et al. 2016; Schulte-Braucks et al. 2019; Sonnentag and Lischetzke 2018).

However, self-conscious emotions and self-esteem are not the only potential outcome variables. First, many negative reactions may occur together; thus, self-conscious emotions often are combined with anger (“humiliated fury”; Tangney and Tracy 2012); multiple affective reactions also are found for disrespectful treatment and social exclusion (Miller 2001; Smart et al. 2009), and the “social pain” associated with social exclusion seems to share neural substrates with physical pain (Eisenberger 2015). Second, given that the concern for self-esteem is so pervasive, people have a wide array of strategies to ward off attacks to the self and restore their self-esteem. Such strategies include affirming the self by derogating others (Fein and Spencer 1997), by focusing one’s attention on valued aspects of the self (Steele 1988), by self-serving attributions (Alicke and Sedikides 2009), or by taking revenge in the form of counterproductive behavior (Schulte-Braucks et al. 2019). Note, however, that attempts to protect the self will not always pay off, and they could be associated with other negative reactions (Schulte-Braucks et al. 2019). As an example, warding off negative feedback by attributing it to a supervisor’s lack of fairness may protect one’s self-esteem but induce anger, sadness, and increased intention to quit. Thus, lower self-esteem is not the only possible consequence of a threat to self-esteem; and it is worth investigating the whole range of outcome variables established in occupational stress research (Sonnentag and Frese 2013).

### Further Research

Although dominated by studies on illegitimate tasks and appreciation, we feel that research on the SOS approach overall has yielded promising results. At the same time, further research is needed, particularly regarding three broad issues: *processes*, *boundary conditions*, and *additional concepts*.

#### Processes

Much of the research on the SOS concept measured only part of the processes postulated to be involved. For example, effects of illegitimate tasks are assumed to be due to a threat to the self; however, self-esteem as an outcome has only been assessed in some of the studies (Eatough et al. 2016; Schulte-Braucks et al. 2019; Semmer et al. 2015; Sonnentag and Lischetzke 2018). Although these studies confirm the important role of self-esteem, there is need for further studies investigating the proposed mechanism, including the role of strategies to ward off the threat, and exploring possible alternative mechanisms that can explain the associations between illegitimate tasks and outcomes.

#### Boundary Conditions

As is true for stressors and resources in general, it is unlikely that the threats and boosts to the self are the same for everyone; thus, individual differences can be boundary conditions for effects. Furthermore, SOS theory focuses on the *meaning* of stressors and resources. It therefore is likely that cultural differences play an important role, as cultures are characterized by “shared basic assumptions, values, and beliefs” (Schneider et al. 2013, p. 362) and thus influence appraisals. In addition to the studies reported above (Ahmed et al. 2018; Munir et al. 2017), further research along this line is needed. Such research should not only focus on “grand” cultural differences (e.g., USA versus India) but should include differences in organizational cultures (for instance: Does the concept of illegitimate tasks make sense to people who have sworn obedience to their superiors, which might imply that, apart from tasks that are illegal, almost any order from these superiors will be perceived as legitimate).

A second boundary condition is the presence or absence of other stressors and resources in the workplace. For example, buffering effects have already been shown for appreciation with regard to illegitimate tasks (Apostel et al. 2018; Dettmers and Biemelt 2018; Pfister et al. 2018), or the effect of interruptions (Stocker et al. 2018). Regarding other stressors, interactions of illegitimate tasks with time pressure have been shown (Schmitt et al. 2015; Zhou et al. 2018).

Finally, individual differences have already been shown in a number of studies, including gender (Omansky et al. 2016); low personal resources such as low trait self-esteem (Eatough et al. 2016), low mental health (Madsen et al. 2014), or low physical health (Kottwitz et al. 2013); attitudes, such as the breadth of one’s role definition (Van Schie et al. 2014), or hostile attributions (Pindek et al. 2018); but also sensitivity towards specific stressors, such as justice sensitivity (Schulte-Braucks et al. 2019).

Such results require replication before we can be sure of their importance. Furthermore, an important question is to what extent interactions are specific or more general: Do stressors and resources interact mainly to the extent that they have implications for the self, als one would assume on the basis of Van den Tooren et al. (2011), or are effects less specific, as suggested by Tesser (2001)?

#### Broader Implications and Additional Concepts

It seems likely that the SOS perspective will lead to additional concepts beyond those investigated so far for several reasons. First, implications for the self are ubiquitous. The concern for a positive self-view is so important that people are likely to scan circumstances and events for implications for the self (notably negative ones), and to do this with a low threshold for noticing such implications. Taking up the argument by Leary and Baumeister (2000, p. 14) that the self-esteem system “should operate continuously (or almost continuously) at an unconscious or preattentive level so that relational devaluation would be detected no matter what else the person is doing” and extending it to personal self-esteem, one could regard the mechanism for detecting threats to the self as a kind of “fire-alarm” that is highly sensitive to signals of danger at any time. As a consequence, threats or boosts to the self are likely to be detected among many other competing stimuli, even if they are rather subtle. A second reason is the fact that threats to the self refer to symbolic meaning. Such meaning is not tied to specific properties of triggers; symbolic meaning may be associated with the size and equipment of one’s office (Greenberg 1988), or with a computer breakdown that is associated with a lack of management concern for providing adequate equipment (Semmer et al. 2007), but also to social behavior, such as incivility (Andersson and Pearson 1999). From this perspective, the possibility to perceive messages as being related to the self is basically endless. As an example, in seminars on continuing education we suspected that the value participants attributed to exchanges with colleagues in similar situations was partly due to an affirmation of the self by seeing people having to deal with similar problems as oneself. Assessing such “exonerating social comparisons” (e.g., “I realized that others have to put on their pants one leg at a time as well”) predicted interest in the course content in a pilot study (Semmer 2012).

One of the most important aspects of SOS is that the concept deals with *meaning related to self* – the meaning attributed to tasks, working conditions, stressors, resources. As shown above, this meaning can be “attached” to any physical property, social behavior, or symbolic act, in addition to any other properties. In that sense, further research in occupational health psychology should examine to what extent effects of stressors and resources are due, for instance, to their energy-related features (e.g., the effort required by stressors conceived as demands, and the effort saved by resources; Bakker and Demerouti 2017), to physical harm (e.g., accidents; physical assaults), but also to what extent they are due to their symbolic meaning, not least in terms of the implications for the self.

## Practical Implications

SOS has a number of practical implications. First, organizations and leaders should be alerted to the importance of messages concerning the self, to their ubiquitous nature, and to the fact that even subtle messages may have an impact. Leaders are more likely to realize the occurrence and effect of strong and obvious messages that offend or affirm the self but less likely to be aware of the impact of more subtle messages (e.g., subtly offending feedback; simple thank-you’s). This is important especially for messages that threaten the self, as negative messages have more impact than positive ones (Leary and Baumeister 2000; Sinclair et al. 2015). We believe that many subtle messages are sent without intention and even without noticing. As an example, the first author observed a meeting where a supervisor said with regard to their IT-system: “Our system is not working well, we need a new one”. Unexpected to the supervisor, this seemingly factual statement turned out to be an offense to those participants who had developed the system years ago. A simple reformulation could have avoided the offense: “The system has worked very well for many years, but now, we have to think about a new development.” There is a danger that the effects of subtle messages are underestimated and that recipients who are offended are regarded as too “thin-skinned”. Alerting managers to this issue in general, and to inform them about concepts such as subtly offending feedback (Krings et al. 2015), about the importance of regularly acknowledging good work (Stocker et al. 2014), of careful inquiry and listening (Van Quaquebeke and Felps 2018), or the nature of illegitimate tasks (Semmer et al. 2015), is of practical importance. This includes the necessity for supervisors to be aware of situations in which they are more likely to communicate in a less careful way (e.g., ego depletion; Barnes et al. 2015).

At the same time, it is important to think about ways of dealing with offenses that do happen; this includes attempts at restoring interactional justice, for instance by explaining why one is acting in a way that might be perceived as inappropriate (Minei et al. 2018) or by apologizing for offenses (Byrne et al., 2014). Ironically, apologies are often avoided because they pose a threat to the self of the person who should apologize (Schumann 2018).

Finally, an important caveat concerns the degree to which it is possible and advisable to avoid offenses to the self at all costs. Sometimes, one needs to convey messages that have offending potential. For instance, sometimes it is necessary to provide negative feedback in order to support the recipient to improve. If the feedback describes serious shortcomings, the recipient’s sense of competence is likely to be threatened. In such a case, delivering the feedback in a fair manner may not help very much; to the contrary, feedback fairness may actually heighten the negative effect on self-evaluation, because recipients have no chance to attribute their failure to anything but their own behavior (Brockner et al. 2003). Shying away from delivering clear feedback in such a situation is rather common (Waung and Highhouse 1997), but that may come at serious costs in terms of missed chances for improvement. Thus, there may be situations in which an anticipated offense may have to be accepted. The main issue is that this is done on the basis of careful and responsible deliberation rather than on the basis of one’s own affective reactions, such as anger and disappointment, fear of conflict, or self-defense.

To be aware of potential implications for the self of one’s subordinates, and to deal with them appropriately, poses additional burdens on supervisors. Not surprisingly, studying cognitions of supervisors who suppressed their tendency to praise subordinates (“praise-hindering cognitions”), Elfering (2016) found that such cognitions were predicted, among other influences, by stressors such as time pressure and illegitimate tasks; together with stressors, these cognitions predicted failure to praise. Training supervisors in dealing with such delicate situations therefore is necessary.

## Final Remarks

We started by suggesting that occupational health psychology should focus more strongly on the content of people’s goals, needs, or motives, as both stressful and positive experiences are related to the extent that people achieve goals or move towards them or away from them. We identified the concern for maintaining a positive personal and social self-esteem as a central motive. Although numerous studies have shown its importance, the concern for self-esteem is not yet a central construct in occupational health psychology. Stress-as-Offense-to-Self theory has put this motive at the center and showed how this perspective can contribute to a better understanding of many issues in occupational health. Despite many questions remaining, we feel that the fresh look on existing phenomena (e.g., appreciation, subtly offending feedback) and the development of new concepts, and the related research, make SOS theory a promising approach. We believe that the scope of phenomena worth being studied from this perspective is far from exhausted. We are looking forward to further work on threats and boosts to the self at work and their effect on health and well-being, the processes involved, the boundary conditions, and the implications for practice.

## Footnotes

1 Interestingly, Hackman and Oldham (1980, p. 72) mention in a footnote that their concept of internal motivation is closely related to work being “self-rewarding”, implying pride in the positive, and shame in the negative case.
